# Maximizing Black applicant matriculation in U.S. PA programs: associations between the number of submitted applications and likelihood of matriculation

**DOI:** 10.1186/s12909-021-02563-5

**Published:** 2021-02-23

**Authors:** Trenton Honda, Trenton D. Henry, Ellen D. Mandel, Alicia Quella, José E. Rodríguez, Shahpar Najmabadi, Virginia L. Valentin

**Affiliations:** 1 Bouvé College of Health Sciences, Northeastern University, Boston, MA USA; 2 Department of Family and Preventive Medicine, University of Utah, Salt Lake City, UT USA; 3 Physician Assistant Program, St. Elizabeth University, Morristown, NJ USA; 4 Department of Physician Assistant Studies, Augsburg University, Minneapolis, MN USA; 5 Office of the Associate Vice President for Health Equity, Diversity, and Inclusion, University of Utah Health, UT Salt Lake City, USA; 6 Division of Physician Assistant Studies Department of Family and Preventive Medicine, University of Utah, UT Salt Lake City, USA

**Keywords:** Physician assistant/associate, Race/ethnicity, Underrepresented minority, Medical education, Matriculation

## Abstract

**Background:**

Physician Assistants (PA) are important members of the medical team, and increasing diversity in healthcare professionals has been consistently associated with improved health outcomes for underrepresented minority patients. In this study of a national cohort of PA program applicants, we investigated whether the number of programs a student applied to (Application Number, AN) was significantly associated with increased likelihood of matriculation into a PA program.

**Methods:**

We examined all applications (*n* = 27,282) to the 2017–2018 admissions cycle of the Central Application Service for Physician Assistants, which is utilized by over 90% of accredited PA programs in the US. As we a priori hypothesized that associations would be non-linear, we used natural cubic splines to estimate the associations between matriculation and AN, controlling for multiple metrics of academic achievement, experience, and applicant demographics. We subsequently used segmented regression analyses (modified poisson regression with robust error variance) to investigate log-linear associations above and below inflection points identified in the spline analyses. Additionally, we explored for effect modification by race/ethnicity.

**Results:**

The strongest associations were observed between application number 2–7, and a threshold effect was observed at > 16 applications, beyond which there was no significant, incremental benefit in matriculation likelihood. Associations differed by race, particularly for application number 2–7, wherein the incremental benefit from each additional application was highest for Black applicants (Likelihood Ratio [LR]: 1.243, 95% CI: 1.136 to 1.360) vs non-Latinx White (LR: 1.098, 95% CI: 1.072 to 1.125), with no additional, incremental benefit beyond 7 program applications. For all other races, significant increased likelihoods of matriculation were observed until 16 program applications.

**Conclusions:**

These findings can help guide pre-PA advisors and PA programs, providing recommended thresholds to applicants on the most cost effective ways to increase their likelihood of admissions, and the PA profession as a whole by providing actionable information that can potentially increase Race/Ethnic diversity in the PA profession and, by extension, medical teams.

## Introduction

Over 50 years ago the Physician Assistant (PA) profession was formed to address the primary care shortage in the United States (US) [1]. During this time the PA profession has grown significantly with over 140,000 licensed PAs and over 250 PA programs to date [2, 3]. This growth has coincided with two national, demographic trends among PA students: A decrease in average age, and an increase in the proportion of women [4].

Admission to a PA program is highly competitive: 2014–2015 data indicates that for every seat there are 2.95 applications in PA compared to 2.43 for medical school [5]. Correspondingly, of the almost 23,000 applications only 34% are matriculated into a PA program [5]. This competitiveness has not decreased over time as the number of PA programs have increased, leading the average PA applicant to apply to six programs with a total cost of $454 to Central Application Service for Physician Assistants in 2020 [6]. This financial investment may be a substantial barrier to some disadvantaged applicants, although previous literature is largely silent on the issue. The only previous, comprehensive study of predictors of matriculation using a national dataset by Yuen and Honda [7] found that underrepresented minority applicants (URMs)—defined as American Indian/Alaska Native, Black or African American, Latinx (Hispanic or Latino) and Pacific Islanders—and older applicants without current graduate record examination (GRE) scores were less likely to matriculate than non-URMs and younger applicants, controlling for the number of programs to which an applicant applied. However, the study did not explore the independent effect of application number on the likelihood of matriculation, and whether the association varied by URM status. It is possible that at least some of the differential effect observed by Yuen and Honda regarding GRE and URM is related to differential access to the GRE exam due to socioeconomic and/or economic barriers. If so, it is likely that the cost of additional CASPA applications would likewise serve as a barrier to students from low SES backgrounds, who tend to be disproportionately URM [7].

The compounding disadvantage of multiple, expensive admissions requirements may help partially explain why applicants accepted to PA programs do not reflect the racial and ethnic diversity of the US population. US Census data indicate that the population is 18.1% Latinx, 13.4% Black or African American, 1.3% American Indian/Alaska Native, and 0.2% Native Hawaiian/Other Pacific Islander [8]. The 2019 NCCPA Statistical Report of Certified PA’s in the US reports that within the PA profession, diversity lags behind that of the US, with 6.6% Latinx, 3.6% Black or African American, 0.4% American Indian/Alaska Native and 0.3% Native Hawaiian/Other Pacific Islander [2, 7, 9]. PA applicants from Black, Latinx, American Indian/Alaska Native, and Pacific Islander groups matriculate at much lower rates than their percentage in the applicant pool, while non-Latinx white applicants matriculate at higher rates than their percentage of the applicant pool. For example, Black applicants make up 6.15% of the applicant pool, but represent only 2.92% of the matriculant pool, while non-Latinx White applicants comprise 65.64% of applicants, and 75.36% of matriculants [5]. Yuen and Honda [7] found a similar disparity in likelihood of matriculation, and found that the disparity between URM and non-Latinx white matriculation became nonsignificant after controlling for academic achievement. This may suggest that systematic disadvantage in, and access to, education and educational resources antecedent to applying may play a significant role in explaining this differential access to PA education they and prior researchers have observed [7].

We hypothesize that the matriculation of students from all backgrounds will be related to the number of programs to which they apply, and that this association will likely have a threshold effect. Further, we hypothesize that the association between application number and matriculation will be impacted by URM status.

## Methods

### Data sources and institutional review board

This is a secondary analysis of a data set including all applicants to the 2017–2018 admissions cycle of the Central Application Service for Physician Assistants, which is utilized by over 90% of accredited national PA programs. Access to the CASPA dataset was provided by the Physician Assistant Education Association (PAEA), and all participant identifying information was removed prior to PAEA providing the data to us for analysis. This research was determined to be exempt (non-human-subjects research) by the University of Utah Institutional Review Board and all methods were carried out in accordance with relevant guidelines and regulations.

### Covariates

The primary predictor of interest was number of applications submitted to various programs by a given applicant (henceforth application number, AN), which was collected as a count variable. In addition, models were adjusted for important potential confounders, including age (continuous), sex (binary: Male; Female), URM status (defined as American Indian/Alaska Native, Black or African American, Latinx (Hispanic or Latino, modeled in a binary fashion: Yes, No), patient care experience hours (5-level categorical variable based on self-reported prior experience), whether the GRE was taken (binary: Yes, No), and overall science grade point average (GPA, modeled as a continuous variable).

### Statistical analysis

Given the extreme range of AN (1 to 118), and its significantly right-skewed distribution, we hypothesized a priori that AN would be non-linearly associated with odds of matriculation, likely with an upward threshold effect beyond which no incremental benefit would be observed. Therefore, we first used natural cubic splines to investigate the association between AN and participants’ likelihood of matriculating into a PA program. We subsequently utilized segmented regression analysis with ψ = 5, yielding cut points for four categories of AN. Subsequently, we used modified Poisson regression with robust error variance [10] to investigate log-linear associations above and below the cut points identified in segmented regression. In secondary analyses, we examined whether the associations between AN and matriculation were modified by applicant race using multiplicative interaction terms.

To visualize predicted probabilities of matriculation in our figures, we created a hypothetical data set for AN of 1 through 50 with every permutation of categorical covariate while holding GPA and age at mean levels of the participant data set; this resulted in 432,000 observations for visualizing predictions.

### Missing data

Our data were highly complete, with only 6.9% of subjects having missingness on any covariate. To ensure that our results were not biased by missing data, we performed multiple imputation with chained equations [11] to generate 50 pooled data sets for conducting regression analysis (resulting in 1.36 million observations). In sensitivity analyses, we repeated our statistical models using complete cases only. All data imputation and statistical analysis were conducted with SAS software version 9.4 [12] and R 3.6.1 [13].

## Results

Table 1 shows the demographics characteristics of our study participants. Out of 27,282 applicants, 8839 (32.4%) matriculated into a PA program. The majority of applicants were female (72.14%), and Non-Latinx White (60.06%), with a mean age of 25.8 years (SD = 5.7, Median = 24). The overall mean AN was 7.3 (SD = 6.1, Median = 6), while 15.4% had an application number of 1. Compared to non-matriculants, matriculants had higher number of programs that they applied to (Median = 8, IQR = 7, versus Median = 5, IQR = 7, P_diff_ < .0001).

**Table 1:** Frequency counts by matriculation status and number of applications [count (%) or mean ± SD]

	Non-matriculated ***n*** = 18,443				Matriculated ***n*** = 8839			
Number of applications	1***n*** = 3448	2–7***n*** = 9008	8–16***n*** = 4954	> 16***n*** = 1033	1***n*** = 742	2–7***n*** = 3350	8–16***n*** = 3790	> 16***n*** = 957
**Age**	28.4 ± 7.61	26.3 ± 5.98	25.0 ± 4.41	25.4 ± 4.31	26.7 ± 6.65	25.0 ± 5.22	24.2 ± 3.70	24.9 ± 3.86
**Sex**								
Female	2432 (70.5)	6386 (70.9)	3594 (72.5)	767 (74.2)	526 (70.9)	2409 (71.9)	2844 (75.0)	724 (75.7)
Male	1007 (29.2)	2616 (29.0)	1357 (27.4)	266 (25.8)	216 (29.1)	936 (27.9)	944 (24.9)	233 (24.3)
**Race**								
NL White	2020 (58.6)	5069 (56.3)	2797 (56.5)	517 (50.0)	562 (75.7)	2349 (70.1)	2551 (67.3)	521 (54.4)
Black	377 (10.9)	945 (10.5)	323 (6.5)	56 (5.4)	35 (4.7)	166 (5.0)	146 (3.9)	35 (3.7)
Latinx	392 (11.4)	1101 (12.2)	526 (10.6)	95 (9.2)	46 (6.2)	311 (9.3)	310 (8.2)	78 (8.2)
Other	428 (12.4)	1318 (14.6)	916 (18.5)	260 (25.2)	61 (8.2)	329 (9.8)	529 (14.0)	239 (25.0)
**Underrepresented minority**^**a**^								
Yes	917 (26.6)	2469 (27.4)	1160 (23.4)	256 (24.8)	109 (14.7)	584 (17.4)	596 (15.7)	196 (20.5)
No	2300 (66.7)	5964 (66.2)	3402 (68.7)	672 (65.1)	595 (80.2)	2571 (76.7)	2940 (77.6)	677 (70.7)

^a^Underrepresented minority includes American Indian/Alaska Native, Black or African American, Latinx and Pacific Islander applicants

**Fig. 1:**
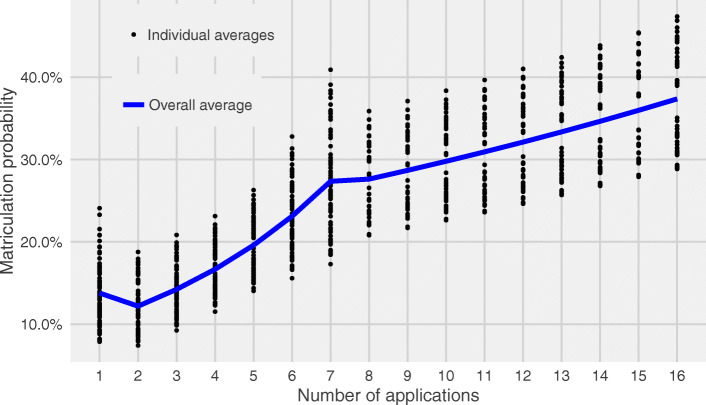
Average predicted matriculation probability by number of applications submitted

Table 2 shows the results of Poisson regression comparing application number to matriculation probability. Inflection points for categories of AN were found to be at 1, 2–7, 8–16, and more than 16 applications (Fig. 1). For ANs of 2–16, each successive application was associated with a statistically significant incremental increase in overall matriculation probability. For 2–7, each additional application is associated with an 11.7% (95% CI: 9.4 to 14.0%) increased likelihood of matriculation; for 8–16, it was 4.3% (95% CI: 2.9 to 5.7%). Underrepresented minority status is associated with a statistically significant disadvantage in matriculation probability (LR 0.623, 95% CI: 0.459, 0.817) at only 1 submitted application, representing a 37.7% reduced likelihood of matriculation compared to non-URM applicants. We investigated for interactions between AN and URM in all other application-number categories and found no significant association; however, when sub-setting URM by its constituent races/ethnicities, we found significant effect modification by race.

**Table 2:** Results of Poisson regression with robust standard errors [likelihood ratio (95% CI)]

	Application number (AN)^**a**^		
	2–7	8–16	> 16
Effect of 1-unit AN increase on matriculation likelihood	**1.117 (1.094 to 1.140)**	**1.043 (1.029 to 1.057)**	1.007 (0.998 to 1.016)

^a^ Adjusted for previous experience, GRE taken, gender, age, GPA, underrepresented minority status

**Fig. 2:**
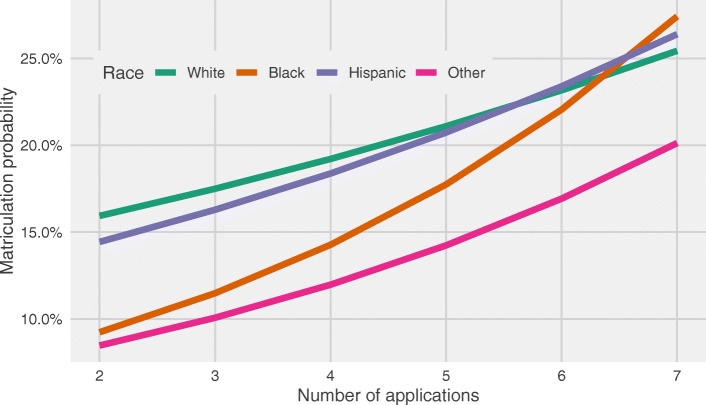
Matriculation probability by race and number of applications for AN 2–7

Table 3 shows the additional independent probability of matriculation that each additional AN provides, stratified by race. Of note, Black applicants have a lower median AN of 4 (IQR: 6) relative to Non-Latinx White applicants’ 6 (IQR: 7), which results in overall Black matriculation of 382 applicants (despite Black applicants comprising 7.6% of applicant pool, this number represents 1.4% matriculated) compared to Non-Latinx White matriculation of 5983 applicants (60.1% of applicant pool, with 21.9% matriculated). Although Non-Latinx white applicants have an initial higher probability of matriculation for lower ANs, other races have higher associations between AN and likelihood of matriculation for higher AN categories. For example, Black, Hispanic, and other races receive 24.3% (95% CI: 13.6, 36.0%), 12.8% (95% CI: 5.4, 20.8%), and 18.9% (95% CI: 11.5, 26.9%) increases in likelihood, respectively, for each additional application when submitting 2–7 applications (Table 3; Fig. 2). For AN 8–16, magnitudes of association become rather homogeneous (Table 3) and, after 16 applications, there is no significant association for AN and matriculation probability for any race, which is consistent with our a priori hypothesis. In sensitivity analyses conducted with complete cases, we found no important differences in our results (data not shown).

**Table 3:** Independent increased matriculation likelihood by race and number of applications submitted [likelihood ratio (95% CI)]

		Application number (AN)^**a**^	
**Race**	**2**–**7**	**8**–**16**	**> 16**
Non Latinx White	**1.098 (1.072 to 1.125)**	**1.041 (1.024 to 1.057)**	1.004 (0.991 to 1.017)
Black	**1.243 (1.136 to 1.360)**	1.054 (0.977 to 1.136)	1.044 (0.993 to 1.098)
Hispanic	**1.128 (1.054 to 1.208)**	**1.050 (1.004 to 1.099)**	1.022 (0.996 to 1.048)
Other	**1.189 (1.115 to 1.269)**	**1.052 (1.017 to 1.088)**	1.004 (0.989 to 1.020)

^a^ Adjusted for previous experience, GRE taken, gender, age, GPA

## Discussion

Our study is the first to demonstrate the association between application number and likelihood of matriculation in a national cohort of PA program applicants. The associations we identified were non-linear, with the strongest associations observed between application number 2–7, and a threshold effect at > 16 applications, beyond which there is no significant incremental benefit in matriculation likelihood. We additionally found that associations differed by race, particularly for application number 2–7, wherein the incremental benefit from each additional application was highest for Black applicants vs non-Latinx White (LR: 1.243 vs 1.098). The increased likelihood of matriculation with the increased number of applications is intuitive but the difference by race is astonishing. In addition, there appears to be no added benefit for Black applicants after application to 7 programs, while all other races continue to increase likelihood of matriculation until 16 applications.

While our finding of an upward threshold effect at > 16 applicants was consistent with our a priori hypothesis, our findings of three distinct associations in the 1–16 AN range was surprising. A number of potential explanations for this exist. First, for applicants with AN = 1, it is likely that many, if not the majority, of these students were applying to programs with special admissions pathways connected to the bachelor’s degree (e.g. 3 + 2 programs or guaranteed matriculation agreements) or due to geographic constraints unique to the applicant (e.g. the only school near spouses place of employment or the applicant’s familial support network). The subsequent precipitous increase in matriculation likelihood for the AN 2–7 increment demonstrates that applicants who apply to more schools, even one more school, dramatically improve their odds of matriculation, while this effect is moderated for AN 8–16. Beyond 16 applications, we found no incremental benefit in likelihood of matriculation.

Importantly, while the likelihood of matriculation is non-linear, the cost for applying to additional schools increases in a linear fashion for applicants, where the costs for CASPA students can be described as follows [6]:

$$ Total\ Cost=179+\left( AN-1\right)55 $$

As a result, the cost to students for each additional application between 2 and 7 is $55, with an associated increased likelihood of matriculation of 11.7% for this investment. However, incremental increases in matriculation likelihood beyond AN 16 is not significantly different from zero, and for Black students, matriculation likelihood does not increase after AN = 7. While the median AN number overall is 6, this finding potentially provides important information for pre-PA student mentors regarding how to guide students in their selection of the number of programs to which it makes sense to apply. Namely, an additional investment of $55 is associated with an increase in matriculation likelihood of over 11%.

We observed that Black applicants had the strongest incremental benefit for additional applications between 2 and 7, with associations nearly 2.5 times higher than for non-Latinx White applicants. However, the median number of programs applied for by Black versus non-Latinx White and Latinx applicants is 33% lower (4 vs 6). Importantly, Black students applying to 7 programs have matriculation likelihoods nearly 73% higher than those who applied for the median (4) for this group. Only one previous study has examined associations between pre-admissions student characteristics and matriculation, and found that URMs and older applicants without current GRE scores were less likely to matriculate than non-URMs and younger applicants [7]. Assuming that the disadvantage Yuen and Honda observed by GRE is, at least in part, due to the economic burden of the GRE requirement, it is possible that our finding of decreased median AN in Black applicants versus non-Latinx White applicants is related to the same underlying disparity in socioeconomic status resulting from the continued presence of systemic anti-Black racism within the United States. Previous studies in medical school applicants have also found financial support to be a strong predictor of medical school applications, and for the impact of low financial support to disproportionately affect underrepresented minorities [14]. This finding may present an important and actionable point for programs that seek to increase diversity in their matriculated class and the PA profession. PA programs can advocate for application fee waivers or fee scholarships for Black and URM students to minimize the financial burden which may be leading to a lower median AN for these groups, thus disadvantaging their likelihood of matriculation. 48.3% of PA programs charge supplemental fees (Median: $50, Range: $20–$300) which may impact the resources available to apply to additional programs. Further, PA applicants designated as coming from an economically disadvantaged background may be advised to be expedient about applying early to capitalize on a limited, first-come, first-served, income based CASPA application fee waiver [6].

Our study has a number of important limitations. First, it is likely that unmeasured confounding is affecting our results, particularly as we were unable to control for objective measures of socioeconomic status. Second, while we observe significant associations between AN and matriculation, we lack information on admission offers. Admission offers are made by programs and reflect a combination of factors such as total number of applicants, logistic, and program factors; therefore, the degree to which AN impacts admissions decisions was unable to be assessed. Further, the matriculation rationale used by any given accepted applicant to a given program is unknown. Third, while our dataset was national, CASPA is not used by every PA program and thus our results may not be applicable to all PA programs. Fourth, our findings for URM applicants may have additional confounding due to the limitations that Deferred Action for Childhood Arrival (DACA) students encounter when trying to finance their education using federal aid programs. This may lead to a substantial number of applications that, despite being provided offers of admission, result in non-matriculation. Last, while our dataset was largely complete, it is possible that the self-reported nature of the data and/or missing race/ethnicity data may bias our results.

These limitations are counterbalanced by a number of important strengths. First, we are using the CASPA dataset, which is the largest dataset on PA applicants and matriculants available, and has important and high quality information on important potential confounders, such as demographics, academic achievement, and prior clinical experience. Second, while we did have some missing data, we used robust missing data imputation techniques which, under assumptions of missing completely at random and missing at random, will yield unbiased results.

## Conclusions

In a national cohort of PA program applicants, the number of programs a student applied to was significantly associated with increased likelihood of matriculation into a PA program. Associations were non-linear, and varied by student race. These findings may help guide pre-PA student mentoring as well as program initiatives to increase diversity by facilitating increased program applications with fee waivers.

## Data Availability

The data that support the findings of this study are available from the Physician Assistant Education Association (PAEA) but restrictions apply to the availability of these data, which were used under license for the current study, and so are not publicly available. Data are however available from the authors upon reasonable request and with permission of PAEA.
